# Overcoming barriers to the adoption of locating technologies in dementia care: a multi-stakeholder focus group study

**DOI:** 10.1186/s12877-021-02323-6

**Published:** 2021-06-21

**Authors:** Silka Dawn Freiesleben, Herlind Megges, Christina Herrmann, Lauri Wessel, Oliver Peters

**Affiliations:** 1 Department of Psychiatry, Charité – Universitätsmedizin Berlin, corporate member of Freie Universität Berlin, Humboldt-Universität zu Berlin, and Berlin Institute of Health (BIH), Lindenberger Weg 80, 13125 Berlin, Germany; 2 German Center for Neurodegenerative Diseases (DZNE), Berlin, Germany; 3 Memory Clinic and Dementia Prevention Center, Experimental and Clinical Research Center (ECRC), Lindenberger Weg 80, 13125 Berlin, Germany; 4 Present address: Federal Ministry for Family Affairs, Senior Citizens, Women and Youth (BMFSFJ), Berlin, Germany; 5 European New School of Digital Studies, European University Viadrina, Große Scharrnstraße 59, 15230 Frankfurt (Oder), Germany

**Keywords:** Alzheimer’s disease, Assistive technology, Adoption, Barriers, Dementia, Focus group, Locating technology, Services, Stakeholders, Surveillance

## Abstract

**Background:**

Locating technologies are a subtype of assistive technology that aim to support persons with dementia by helping manage spatial orientation impairments and provide aid to care partners by intervening when necessary. Although a variety of locating devices are commercially available, their adoption has remained low in the past years. Several studies have explored barriers to the adoption of assistive technologies from the perspective of professional stakeholders, but in-depth explorations for locating technologies are sparse. Additionally, the inputs of business professionals are lacking. The aim of this study was to expand knowledge on barriers to the adoption of locating technologies from a multi-stakeholder professional perspective, and to explore strategies to optimize adoption.

**Methods:**

In total, 22 professionals working in business (*n* = 7), healthcare (*n* = 6) and research (*n* = 9) fields related to gerontology and gerontechnology participated in our focus group study. Perceptions on the value of using locating technologies for dementia care, barriers to their adoption, as well as salient services and information dissemination strategies were explored. After verbatim transcription, transcripts were analysed following an inductive data-driven content analysis approach in MAXQDA.

**Results:**

Six key adoption barriers centering on: (1) awareness-, (2) technological-, (3) product characteristic- and (4) capital investment-based limitations, (5) unclear benefits, as well as (6) ethical concerns emerged. The interplay between barriers was high. Five core themes on services and information dissemination strategies centering on: (1) digital autonomy support, (2) emergency support, (3) information dissemination actors, (4) product acquisition, and (5) product advertising were extracted.

**Conclusions:**

Our study with interdisciplinary stakeholders expands knowledge on barriers to the adoption of locating technologies for dementia care, and reinforces recommendations that an interdisciplinary strategy is needed to optimize adoption. Also, our findings show that focusing on services to increase digital autonomy and on information dissemination strategies has been largely overlooked and may be particularly effective.

**Supplementary Information:**

The online version contains supplementary material available at 10.1186/s12877-021-02323-6.

## Background

The development and deployment of assistive technologies (ATs) represents an opportunity to reshape dementia care on a global and socioeconomically diverse scale at potentially low costs [1]. Several types of ATs exist to compensate for a multitude of cognitive and physical deficits in persons with Alzheimer’s disease (AD) and related dementias [2]. In the literature, locating, or ‘monitoring’, ‘surveillance’, ‘tracking’, or ‘wayfinding’ ATs that use satellite-based positioning technology such as global position systems (GPS) have received considerable attention [3, 4]. Indeed, spatial orientation impairments in AD develop early [5], are common [6, 7], and can cause significant stress and burden for persons with dementia and their care partners [8]. Prevalence rates of persons with dementia getting lost even in familiar environments range from 17% [6] to 75% [7] depending on definitions and reporting measures used, which exposes persons with dementia to risks that can result in life-threatening circumstances [9]. To avoid such risks, care partners often limit independent outdoor ambulation by opting for chaperon, sedative or incarceration-type prevention measures [10] although these measures can negatively impact biopsychosocial health [11]. By contrast, locating devices can promote the independence and safety of persons with dementia by helping manage spatial orientation impairments and by supporting care partners to intervene when necessary [12].

To date, acceptability studies with different stakeholders including persons with dementia and care partners (hereafter end-users), as well as with healthcare and research professionals report favorable perceptions on the appropriateness and openness of using locating technologies for dementia care [13, 14]. Yet, outside research environments, end-users generally have little awareness of the existence of these technologies [15, 16]. Consequently, adoption rates remain low [17, 18] despite the increasing availability of commercial products [12]. Similar findings have been reported for the broader category of ATs [15], which has resulted in the exploration of adoption barriers by these stakeholders [13, 14, 19]. Predominant ATs adoption barriers include awareness-, translational-, effectiveness-, ethical-, and structural-based barriers [14], as well as cost factors [20]. However, very few studies have specifically focused on locating technologies. As such, key factors affecting their adoption might be overlooked [21]. For example, to our knowledge at least one community-based Norwegian study has examined the factors affecting the successful deployment of GPS technologies for public dementia care services, and reports that early adoption in the course of AD is critical [22]. Early adoption has also been highlighted as a central factor affecting product usability and long-term adoption in a Canadian-based study [23]. Certainly, additional studies in different cultural backgrounds are warranted. Also, successful adoption might not strongly depend on early intervention for other ATs.

In addition, existing studies have largely overlooked the voices of business stakeholders intimately involved in product design, development, and commercialization although their inclusion has been recommended [24]. The low involvement of business professionals in past research could help explain the paucity of recommendations on which services [25] should be offered to end-users and which information dissemination approaches such as marketing strategies [26] should be utilized to help maximize end-user awareness, positive user experience (UX), and product adoption [26, 27]. Examples of services such as customer support including product training and technical support in emergency situations have been identified as central needs by end-users [19, 28]. In research settings, these needs are also recognized by researchers as end-users typically receive study-developed product manuals and product training. Still, it remains unclear how to effectively reach and support end-users outside research settings.

Using insights from focus groups with key stakeholders from business, healthcare and research fields, the current study complements existing research by providing an in-depth exploration of the adoption barriers, and recommendations for salient services and information dissemination strategies for locating technologies for dementia care. The goal was to identify ways to optimize the adoption of locating technologies for end-users.

## Methods

### Participants and setting

We utilised a purposive sampling technique to recruit professionals working in fields related to gerontology and gerontechnology from our work network. In total, seventy professionals were contacted via personalized e-mail to partake in a half-day focus group study held at the Memory Clinic of the Charité Universitätsmedin Berlin, Germany, in May 2016. Invitations outlined the study purpose, methodology and organizational details. Specifically, professionals were from business fields within the technology industry sector (representatives of ATs companies with current gerontechnology focus including company executives and executive associates, marketing analysts, UX designers, and software developers), healthcare fields (representatives of Alzheimer societies, community organisations serving older adults with disabilities and nursing homes including local community representatives, managing directors, healthcare managers, social workers, gerontologist, as well as education and program coordinators), as well as research fields (research associates, project managers, group leaders, as well as postdoctoral and doctoral researchers from the fields of gerontology, rehabilitation sciences, social work, health services administration, medical sociology and rehabilitation science, nursing sciences, and gerontechnology). To maximize group homogeneity and interaction [29], professionals were separated into groups based on their professional field. We estimated that sample sizes per group of approximately ten to fifteen would be sufficient to reach data saturation based on sample homogeneity [29]. All professionals who participated provided their written informed consent, and the ethics committee of the medical faculty of the Charité Universitätsmedizin Berlin approved of the study (protocol number EA4/033/16). Participation was voluntary, there were no exclusion criteria, and no incentives for participation were provided. To help ensure that professionals felt comfortable when sharing their thoughts and experiences, discussion moderators indicated that each participant would be given a unique identification number known only to the research team when coding raw data. All methods were carried out in accordance with relevant guidelines and regulations.

### Study design

A qualitative study in the form of focus groups to obtain information from various viewpoints [30] was performed. To inform the study design, we employed a qualitative description methodology [31]. Qualitative description is particularly helpful to gain an in-depth understanding of healthcare-related topics and useful “because of its ability to provide clear information on how to improve practice” (p. 2) [31]. To identify topics and structure the focus group, an interview guide based on a review of the relevant literature was developed [13–20, 28, 30–32]. The final guide comprised of three sections detailed below. Each group was led by a discussion moderator (HM, OP, LW) and one or two assistant moderators (SDF, VL, RS, GÖ, FK) who kept notes and audio recorded the discussion. All moderators and assistant moderators were provided with the interview guide prior to the focus groups. Also, a dry run of the interview guide was performed to allow for adjustments in wording or placement of questions, and to ensure familiarity and consistency with the guide between groups. Focus groups lasted approximately three hours, which included the administration of informed consent and filling out of questionnaires.

#### Section 1: exploration of perceptions on value of use

Professionals first filled out a standard demographic questionnaire which also assessed years of experience (i.e., professional or personal) with dementia and ATs, as well as one-time and monthly pay willingness for a locating device from the perspective of end-users (i.e., proxy measurement). Also, technological affinity was assessed with the Technological Affinity for Electronic Products questionnaire (TA-EG; Likert scale 19–95, scores proportional to affinity) [33]. The TA-EG assesses key aspects of the technology acceptance model which provides information on technology acceptance and use [34]. Then, professionals’ perceptions on the value of using locating technologies for dementia care were explored by having them write down and discuss at least two keywords or phrases they associate with their use. Exploring perceptions served as an icebreaker [32] to allow professionals to acclimatise to their group before moving onto the next sections.

#### Section 2: exploration of adoption barriers

Thereafter, obstacles to the adoption of locating technologies by end-users were explored by examining views on personal experience, product characteristics, and clinical needs and expectations. To supplement the discussion, a GPS watch marketed for persons with orientation impairments and a smartphone with a pre-installed native android application to locate the watch were presented. These products were available due to our concurrent UX study with persons with dementia and care partners [12], and are displayed in more detail [see Additional file 1].

#### Section 3: exploration of services and information dissemination strategies

Lastly, views on salient services and information dissemination strategies, including recommendations for customer services, service provision methods, and promotional methods such as product advertising were discussed. To supplement the discussion, the flyers of two commercially available GPS watches marketed for persons with orientation impairments [12], which included the GPS watch shown in the previous section, were presented.

### Data analysis

Audio data were digitally recorded, and transcribed verbatim (SDF, HM, CH) into MAXQDA [35]. Afterwards, transcripts were cross-checked with the recordings to ensure validity (SDF, HM, CH). Transcripts were thematically analysed (SDF, HM, CH) using content analysis that followed a data-driven inductive data analysis method [36]. First, common patterns and themes were independently identified and chunked into thematic codes while staying as close to the transcripts as possible. Any unclear quotes and counter-arguments were also noted. Then, codes were further refined into subthemes, and lastly discussed during multiple meetings until team consensus (SDF, HM, CH) was reached to ensure reliability. Data saturation was also assumed to be reached, as no new information could be added to the codes. All authors reviewed and discussed the final codes.

Written perceptions from the first section of the focus groups provided the opportunity to obtain opinions from all participants. As such, a theme density (i.e., number of times a theme arose), supplemented by contributions in the open discussion, was calculated for this section. Reporting of qualitative data using the COREQ (COnsolidated criteria for REporting Qualitative research) Checklist [37] is displayed in more detail [see Additional file 2]. Quantitative data were analysed by performing descriptive statistics. Potential group differences based on group membership or gender were compared with Kruskal Wallis or Mann-Whitney tests, as appropriate. Statistical significance was set at *P*-value < 0.05 using IBM SPSS Statistics, version 23 [38].

## Results

### Participant characteristics

In total, 22 professionals out of the 70 contacted participated (*n* = 35, no response; *n* = 8, unavailable; *n* = 5, no-show). The final groups were: (i) business (*n* = 7, with n = 3 company executives, *n* = 1 executive associate, *n =* 1 software developer, *n =* 1 marketing analyst, and *n =* 1 UX designer) (ii) healthcare (*n =* 6, with *n =* 1 social worker, *n =* 1 gerontologist, n = 2 managing directors of AD societies, and *n* = 2 healthcare managers of community organisation serving older adults with disabilities), and (iii) research (*n* = 9, with *n =* 1 postdoc (gerontology), *n =* 3 PhD students (gerontology, rehabilitation sciences, and medical sociology and rehabilitation science), *n =* 3 project managers (health services administration, and two gerontology), and *n =* 2 research associates (both gerontechnology). No significant group membership differences were found. One significant difference between gender and dementia experience was found, Mann-Whitney *U* = 20.500, z = − 2.681, *P* = .007, with a mean rank of 7.55 for males and 14.79 for females. Participant demographics are presented in Table 1.

**Table 1:** Descriptive characteristics of the participants

Variables	Business (*n* = 7)	Healthcare (*n* = 6)	Research (*n* = 9)	All (*n* = 22)^a^
Age	44.3 ± 10 [32–55]	46.7 ± 12.9 [28–62]	37.1 ± 11.1 [27–62]	42 ± 11.5 [27–62]
Gender, m/f (female)	5/2 (29)	2/4 (67)	3/6 (67)	10/12 (55)
Education, n				
High school	1 (14)	1 (17)	–	2 (9)
College	1 (14)	1 (17)	–	2 (9)
Any university^b^	5 (71)	4 (67)	9 (100)	18 (82)
Dementia exp. yrs., n				
< 2 yrs	1 (14)	–	4 (44)	5 (23)
2–5 yrs	4 (57)	1 (17)	–	5 (23)
5–10 yrs	2 (29)	2 (33)	2 (22)	6 (27)
> 10 yrs	–	3 (50)	3 (33)	6 (27)
ATs exp. yrs., n				
< 2 yrs	1 (14)	1 (17)	4 (44)	6 (27)
2–5 yrs	3 (43)	2 (33)	1 (11)	6 (27)
5–10 yrs	3 (43)	2 (33)	4 (44)	9 (41)
> 10 yrs	–	1 (17)		1 (5)
Pay willingness, once	235.6 ± 134.6 [99–500]	255 ± 193.8 [100–600]	211.7 ± 176.6 [20–500]	231.1 ± 162.3 [20–600]
Pay willingness, monthly	16.3 ± 8.2 [5–30]	20.5 ± 8.1 [10–30]	17.9 ± 17.6 [0–50]	18.1 ± 12.5 [0–50]
TA-EG (range 19–95)	76.6 ± 8.7 [59–83]	64.7 ± 6.7 [58–74]	68 ± 9.9 [57–83]	69.7 ± 9.7 [54–83]

*Abbreviations*: *ATs* assistive technologies, *exp.* experience, *m/f* male/female, *n* number, *TA-EG* Technological Affinity for Electronic Products, *yrs.* years

Continuous and discrete variables are displayed as mean ± standard deviation [range].

Standard deviations are rounded to nearest decimal point. Percentages are rounded to nearest whole number. TA-EG scores are proportional to technological affinity. Pay willingness in Euros.

^a^Business group: *n* = 3 company executives, *n* = 1 executive associate, *n =* 1 software developer, *n =* 1 marketing analyst, and *n =* 1 UX designer; healthcare group: *n =* 1 social worker, *n =* 1 gerontologist, *n =* 2 managing directors of AD societies, and *n =* 2 healthcare managers of community organisation serving older adults with disabilities; research group: *n =* 1 postdoc (gerontology), *n =* 3 PhD students (gerontology, rehabilitation sciences, and medical sociology and rehabilitation science), *n =* 3 project managers (health services administration, and two gerontology), and *n =* 2 research associates (both gerontechnology).

^b^For education, “any university”: one business, healthcare and research professional, respectively, obtained a Master’s degree, and four business, three healthcare and eight research professionals obtained a PhD degree.

### Perceptions of value of use

We identified three recurrent themes on perceptions on value of use and nine subthemes, displayed in Table 2, Section 1. No differences in theme density between groups were found. The shared perception was that using locating technologies could result in increasing end-users’ quality of life on psychological, social, and physical levels by: (i) promoting the personal security and (ii) independence of persons with dementia, and by (iii) reducing stress and burden experienced by care partners. These benefits could be achieved due to location finding, risk reduction, supporting autonomous mobility and social engagement, by offering peace of mind for care partners by assisting with remote location, and by improving caregiving resource utilization.

**Table 2:** Overview of themes and subthemes illustrated with quotes per focus group section

**Section 1: Exploration of perceptions on value of use**			
**Themes**	**Subthemes**	**Illustrative quotes**	**Theme density (%)**^a^
Promote security for persons with dementia	• Location finding	P2, Business, Company executive: *“Security is guaranteed by the product since for example, when persons with dementia do not come home at a specific time, they can be located.”*	n_b_ = 5 (71) n_h_ = 3 (50) n_r_ = 6 (67)
	• Risk reduction	P2, Business, Company executive: *“A lot of people have been saved with these products from freezing, drowning,* etc.*”*	
*Counterargument*	• *False sense of security*	P15, Research, PhD student (gerontology)*: “I can see with the app where a person with dementia is, on which street corner, but I can’t see whether s/he is crossing at a red light or not.”*	*n*_*b*_ *= 0 (0)* *n*_*h*_ *= 0 (0)* *n*_*r*_ *= 1 (11)*
Promote independence for persons with dementia	• Autonomous mobility	P15, Research, PhD student (gerontology): *“These products can also help maintain or increase the freedom of movement and independence of persons with dementia.”* P6, Business, Company executive: *“If you don’t have such a system, then you have someone telling persons with dementia: “Stop” Stay put! Where are you going again?”*	n_b_ = 3 (43) n_h_ = 4 (67) n_r_ = 6 (67)
	• Social engagement	P15, Research, PhD student (gerontology): *“Yeah I mean like when you can see a daily profile of persons with dementia—where one likes to go, spend their time, what they find interesting in their neighbourhood.”*	
*Counterargument*	• *Feeling tracked*	P5, Business, Marketing analyst*: “The persons that wears the product can feel like they are being tracked, and that’s not a good feeling.*	*n*_*b*_ *= 2 (29)* *n*_*h*_ *= 2 (33)* *n*_*r*_ *= 2 (22)*
Reduce stress and burden for care partners *Counterargument*	• Assistance with remote location • Efficient resource utilization • *Uneasiness about tracking*	P2, Business, Company executive: *“It makes me feel more secure because I’m worried that my [fictitious] dad might not find his way back home although he might be able to… We have clients come up to us and say: ‘Thank you, thank you, thank you! We can let our father, uncle,* etc. *walk alone again.”* P6, Business, Company executive: *I see monitoring also positively. There are a lot of people in professional care settings or care partners who feel responsible in providing this monitoring.”* P22, Research, Research associate (gerontechnology): *“To make it easier to care for persons with dementia… It might be more comfortable for formal care settings because they can save on personnel or invest less time in these [locating] task.”* P5, Business, Marketing analyst*: “But also care partners that use the product can feel uneasy because they are tracking persons with dementia.”*	n_b_ = 2 (29) n_h_ = 2 (33) n_r_ = 5 (56) *n*_*b*_ *= 1 (14)* *n*_*h*_ *= 0 (0)* *n*_*r*_ *= 0 (0)*
**Section 2: Exploration of adoption barriers**			
**Themes**	**Subthemes**	**Illustrative quotes**	
Awareness limitations	• Low knowledge transfers between stakeholders • Limited information on, and access to commercial products • Low technological affinity of end-users	P14, Research, Postdoc (gerontology): *“I can’t use what I don’t know exists. That’s the main problem I learned after conducting 105 interviews [with persons with dementia and care partners].”* P11, Healthcare, Managing director of AD society: *“From the perspective of end-users, this is a product that I don’t know, that is unfamiliar… Product awareness is still largely inadequate.”* P2, Business, Company executive: *“My personal opinion: Way too early. End-users don’t know that these products exist.”* P21, Research, Research associate (gerontechnology): *“General practitioners don’t have an overview of all commercially available products. The same goes for nursing facilities.”* P4, Business, Software developer: “*If care partners need it [GPS technology], where do they go? Where can you buy it? You won’t find it in a supermarket or media store! You first have to research it and if you’re not from this line of work, it’s hard [to find information].”* P14, Research, Postdoc (gerontology): *“Some [persons with dementia and care partners] say: ‘I’ve read or heard about this, but I don’t know where I can buy these products. I guess online’.”* P18, Research, Project manager (gerontology): *“There are certainly older adults that are good with technologies, which have smartphones. But there are some older adults that have no experience—that are technology skeptic.”* P13, Healthcare, Healthcare manager: *“We are talking about the age group 70 plus, right? The next generation will be more familiar with these technologies.”*	
Technological limitations	• Unsatisfactory reliability and accuracy of location function • Limited functionality	P16, Research, PhD student (rehabilitation science): *“If it’s in the name, it has to work!”* P14, Research, Postdoc (gerontology): *“If it [location] doesn’t work reliably, it won’t reduce care partners stress and burden.”* P22, Research, Research associate (gerontechnology): *“When one enters an underground parking lot or a building, then you can often pretty much forget about location. The product has to be more than 150% reliable. If not, you can forget it!”* P22, Research, Research associate (gerontechnology): *“It should be low maintenance… You should be able to locate immediately, without having to wait for updates. And if there’s a discrepancy of a few meters and I’m in the pedestrian zone and there are a lot of people around, it could be that I don’t find someone who is two meters away.”* P16, Research, PhD (rehabilitation science): *“The location of two minutes ago might not be valid.”* P11, Healthcare, Managing director of AD society: *“For the battery, there’s a signal notifying you when you are running low on power. Of course, the question is when you receive a notification. Because it’s totally annoying if the product starts to beep when you are alone. This might lead to more disorientation.”* P5, Business, Marketing analyst: *“Geofencing is one aspect. I would program other intelligent functions, such as integrated temperature recognition. There are maybe other things at persons with dementias’ location that could active an alarm. So I would program intelligent systems.”* P7, Business, UX designer: *“I would like a product that notifies me when my [fictitious] mom leaves her home without the product.”*	
		P22, Research, Research associate (gerontechnology): *“And then an emergency recognition, so that when persons with dementia fall down or stumble on something, that the system recognizes this.*	
	• Poor battery performance	P3, Business, Executive associate: *“If I need a GPS product all day, maybe it won’t last all day. And cellphones [for care partners] either.”*	
		P1, Business, Company executive: *“How long does the battery last? Since our latest update, max two days…”* [P4, Business, Software developer: *“Max? Yeah, that’s a problem.”*]	
Product characteristic limitations	• Low end-user focus and product customizability in product development	P10, Healthcare, Managing director of AD society: *“A decisive factor is how easy the different functions are to understand… Regarding product functions, less is more.”* P12, Healthcare, Healthcare manager: *“There is a person with dementia who lives in our nursing facility. He doesn’t leave the grounds without his fanny pack. If you could put the product in his fanny pack and it would still work, that* *would be ideal.”* P16, Research, PhD (rehabilitation science): *“Individual configuration... to set up a custom area ‘from this crossroad to here’.”* P9, Healthcare, Gerontologist: *“I find it good that there are different functions, such as the emergency and two-way communication. But these functions should be individually customizable, looking at actual severity level and other factors.”* P3, Business, Executive associate: *“What dementia severity does the person have? A one-size-fits-all product won’t work.”* P9, Healthcare, Gerontologist: *“Persons developing the technology don’t involve end-users. First of all to ask: ‘What do you want from your product? What should the product look like?’”* P2, Business, Company executive: *“Persons with dementia and care partners are not our primary market group.”* P6, Business, Company executive: *“I think I’ve realized that we have to think a lot more from the perspective of end-users. This should always be the starting point and then think about hardware and so forth.”*	
	• Unsatisfactory and stigmatizing aesthetics	P11, Healthcare, Managing director of AD society: *“Most products are not aesthetically pleasing for females.”* P10, Healthcare, Managing director of AD society: *“For the design, yeah, there’s black, but I would think of offering products in more colors. This should be possible.”* P1, Business, Company executive: *“The products are too big! We would gladly reduce the size if the technology would allow it… The problem is that you need space for a better battery, for power, for… And so that it’s comfortable to wear, particularly if it’s to be worn on the wrist.”* P1, Business, Company executive: *“Some products have security straps. But no one wants to walk around with such a thing!”*	
	• Product costs	P1, Business, Company executive: *“The biggest barrier is always the price.”* P10, Healthcare, Managing director of AD society: *“There’s a cost problem at the moment. Can I afford this? Are there any additional costs once I use it? Products are simply too expensive.”* P8, Healthcare, Social worker: *“Cost is a big factor. Do I purchase it or not for the last phase of my life?”*	
Capital investment limitations	• Lack of funding	P1, Business, Company executive: *“There’s no one here [in the other groups] that I know was involved in product development, right? There’s a big discrepancy. We could all say how products should be and what could be done. But you have to have the money to do this…you first have to have the money to invest.”*	
	• Low product development follow-through	P1, Business, Company executive: *“There are too many products that are not developed to the end.”*	
Unclear benefits	• Unclear perceived need by • end-users	P11, Healthcare, Managing director of AD society: *“Persons with dementia do not see that they need it [locating device]. At most, care partners recognize a need.”* P6, Business, Company executive: *“I can imagine that my fictitious father might need such a product. But whether he sees a need? There might be no recognized need.”* P2, Business, Company executive: *“No end-user purchases it out of prevention. All buy it because something has already happened.”*	
		P8, Healthcare, Social worker: *“It [using a locating technology] of course depends on dementia severity.*	
	• Reliance on other trusted locating methods	P5, Business, Marketing analyst: *“I [care partners] might pragmatically get more involved with the [local] community.”*	
	• Lack of studies and unclear clinical effectiveness	P21, Research, Research associate (gerontechnology): *“End-users should be more involved [in research and development]. They should test products and then we will better understand what needs to be improved.”* P1, Business, Company executive: *“We need the Charité and the German Alzheimer Society to come out with studies. Then there will be a bigger discussion.”* P2, Business, Company executive: *“There are dementia severities, and then it’s always the question: ‘How long can I [care partners] let persons with dementia move about and use the product [without studies with persons with dementia with different AD severities]?”* P19, Research, Project manager (gerontology): *“I can’t evaluate products as a lay person. Do I need it? Does it work?”*	
	• Previous negative user experience	P1, Business, Company executive: *“The technology is constantly changing. And those [end-users] who did test it three-four years ago… they had bad experiences. And if it doesn’t work on the first attempt: Next! Forget it!”*	
Ethical concerns	• Balance between autonomous mobility of persons with dementia and control by care partners • Unclear information on data privacy and security	P19, Research, Project manager (gerontology): *“As a person with dementia, I have my autonomy, I have my rights. I might not know that I am being located at a particular time. But for care partners, that’s really not a problem because they have a sense of security. There’s a big difference between medical professionals and care partners, where medical professionals say: ‘That’s an infringement on personal freedom’, and care partners say: ‘I don’t care. I have to know where [person with dementia] is!’”* P13, Healthcare, Healthcare manager: *“There are also data security aspects, so basically the fear of being watched or controlled.”* P3, Business, Executive associate: *“I think of tracking firms that collect large amounts of data, secretly collecting information on movement profiles… Do we reduce independence or increase security?”*	
• Unclear legal rights on location of others		P6, Business, Company executive: *“We are very involved with this at the moment. How many movement profiles can be programmed and saved, under which conditions,* etc.*? This is a very difficult situation at the moment for all businesses involved.”* P15, Research, PhD student (gerontology): *“There are a lot of decisions at the moment on what is allowed regarding locating others.”* P2, Business, Company executive: *“Ultimately, it’s a legal problem with too many unknowns. Are we allowed to do this, to do that? This hinders commercialization. First get approval from a court of law. The external framework could be better. This is one of the main reasons why it [GPS technologies] has not spread so quickly.”*	
**Section 3: Exploration of services and information dissemination strategies**			
**Themes**	**Subthemes**	**Illustrative quotes**	
Digital autonomy support	• Installation and product training support	P15, Research, PhD student (gerontology): *“A support that’s really tailored to end-users. Particularly to help set up and configure the product.”*	
		P3, Business, Executive associate: *“If I use it for the first time, I would like to have an installation assistance on how to use the app that I can maybe turn on and off.”* P17, Research, Project manager (health services administration): *“That you really have an on-location support that also makes house calls to help one get started with the product.”* P5, Business, Marketing analyst: *“Case-management service support… If I have a person with deficits, with a certain problem severity, then I can also offer other attractive service support features.”*	
	• Automated technical support	P6, Business, Company executive: *“…for example, that telephone numbers are listed on a website, that frequent questions such as ‘How to install the program’,* etc. *are provided.”*	
*Counterargument*	• *Unclear affordability of services for end-users*	P2, Business, Company executive*: “But these services have to be affordable and there are simply too many older adults that do not have the financial capacity.”*	
Emergency support	• Emergency call centers	P10, Healthcare, Managing director of AD society: *“At a minimum [for emergency situations], there has to be a hotline.”* P17, Research, Project manager (health services administration): *“It’s important to have an emergency support call service that answers whatever question you might have.”*	
*Counterargument*	• *Lack of personnel and financial resources*	P6, Business, Company executive: *“When an alarm is set out, because you have personnel changes every 24 h, you have to have a lot of people that do this [job]. Who does it on the weekends?”* P22, Research, Research associate (gerontechnology): *“Support that is available 24/7…but this has to be financed. That’s also really expensive!”*	
Information dissemination actors	• Multi-actor approach: memory clinics, medical supply stores, general practitioners, local government, and healthcare insurance companies	P9, Healthcare, Gerontologist: *“You could involve memory clinics.”* P?, Healthcare: *“It would be really easy to involve medical supply stores.”* P17, Research, Project manager (health services administration): “*I think that general practitioners should be involved because they are typically the starting point. There’s a trust-based relationship there.”* P19, Research, Project manager (gerontology): *“There’s a pilot project in [German city], where the government has set up a counselling center also for technology for older adults…They can advise you there…You can go to them, but they can also go to you.”*	
		P5, Business, Marketing analyst: *“What we need is support from an established healthcare insurancecompany that creates a ‘service-support platform’.”*	
*Counterargument*	• *Financial, time and lack of follow-up limitations of proposed actors*	P9, Healthcare, Gerontologist: *“But persons with dementia come here [memory clinic] at max every six months…”* P22, Research, Research associate (gerontechnology): *“If my general practitioner talks to me about such products, I’d feel like they are trying to sell me something. I don’t go to my general practitioner for that.”* P19, Research, Project manager (gerontology): *“GPs are saying: ‘What else are we also supposed to do?’ Who pays for this extra work?”*	
Product acquisition	• Retail options	P19, Research, Project manager (gerontology): *“At the moment, most products can be bought online. So there’s a lack of vendors with whom older adults can talk to. I think personal talks are extremely important.”*	
	• Trial periods	P8, Healthcare, Social worker: “I might see an ad for such a product and think: ‘Oh, that’s cool!’ But I still have no experience with the product. Experience is elementary. If I don’t have experience, I won’t use the product.” P12, Healthcare, Healthcare manager: *“For me, it would be a requirement that I can test the product first for two to three weeks without having to pay a big amount for this. Maybe a little fee, but not the entire amount.”* P16, Research, PhD (rehabilitation science): *“For many, it’s important to be able to experience the product, to touch it, feel it. Maybe offer a trial purchase.”*	
	• Government subsidies	P5, Business, Marketing analyst: *“In nursing care, there are a lot of government care grants…different financial plans, how you can use these various services.”*	
Product advertising	• Promotion of independence and autonomy	P2, Business, Company executive: *“We are trying to erase the word tracking.”* P3, Business, Executive associate: *“We’ve replaced the word tracking with guardian angel.”*	
		P10, Healthcare, Managing director of AD society (flyer-feedback): “*And particularly in old age, the importance of remaining independent without sacrificing comfort and safety.”* P11, Healthcare, Managing director of AD society (flyer-feedback): *“I prefer the description on this flyer. It’s simple and contains all you need to know. I see security, quality of life, liberty. The visual presentation is good, and the font size is nice and large. This other flyer is not directed toward persons with dementia, but rather only toward care partners.”* P19, Research, Project manager (gerontology; flyer feedback): *“This picture is a no-no for the current generation of older adults.”*	
	• Seal of quality from trusted organisations	P1, Business, Company executive: *“There have to be institutions. That’s why I’m here today… In the end, the Charité or similar is missing. The stamp from ISO does not suffice. When Charité or German Healthcare Ministry is visible, then there’s a completely different quality level that is achieved.”*	
	• Addressing concerns of end-users: data security, product characteristics, and service details	P20, Research, PhD (medical sociology and rehabilitation science): *“It could be a marketing problem… for example, that it’s not clear that it can be avoided that everyone sees my data and locate me. If I don’t know that, I don’t buy it.”* P10, Healthcare, Managing director of AD society: *“This aspect [data security] has to be covered in product advertising.”*	
		P16, Research, PhD (rehabilitation science): *“I really think that there is a general lack of clear information on data security. It’s really important that data security is communicated and mentioned and that it’s theoretically possible for a third party to access data sensitive information. So that people know what to do in such situations.”* P5, Business, Marketing analyst (flyer feedback): *“What I still don’t know is whether I have to take the watch off every day and charge it.”* P6, Business, Company executive: *“Let’s say I receive a message at 4 am about my mother and this happens three nights in a row. I’ll be woken up and I can’t really help… What happens then?”*	
	• Conventional advertising platforms: television, magazines, pharmacies	P14, Research, Postdoc (gerontology): *“There are probably people that don’t check online for this [GPS product], but rather watch TV. So maybe use TV ads to multiply information.”* P22, Research, Research associate (gerontechnology): *“I saw an ad in [free magazine with large older adult readership] about a high blood pressure product. I thought that was really good. A magazine that a lot of older adults read—not just persons with dementia and care partners. And the magazines are free. You can just take one.”* P14, Research, Postdoc (gerontology): *“Maybe there should just be ads placed in pharmacy windows.”*	
*Counterargument*	• *Financial limitations*	P1, Business, Company executive*: “I don’t produce million-dollar TV ads.”*	

*Abbreviations*: *n* number; *n*_*b*_ business, *n*_*h*_ healthcare, *n*_*r*_ research

Percentages in parentheses rounded to nearest whole number

^a^Theme density calculated based on professionals’ written keywords or phrases and supplemented by contributions in the open discussion

P2, Business, Company executive: *“A lot of people have been saved with these products from freezing, drowning, etc.”*

P15, Research, PhD student (gerontology): *“Yeah I mean like when you can see a daily profile of persons with dementia—where one likes to go, spend their time, what they find interesting in their neighbourhood.”*

P2, Business, Company executive: *“It makes me feel more secure because I’m worried that my [fictitious] dad might not find his way back home although he might be able to … We have clients come up to us and say: ‘Thank you, thank you, thank you! We can let our father, uncle, etc. walk alone again.”*

P22, Research, Research associate (gerontechnology): *“To make it easier to care for persons with dementia … It might be more comfortable for formal care settings because they can save on personnel or invest less time in these [locating] task.”*

Still, professionals expressed mixed feelings for each perceived benefit. In particular, products could represent a sense of false security due to inaccurate location.

P15, Research, PhD student (gerontology)*: “I can see with the app where a person with dementia is, on which street corner, but I can’t see whether s/he is crossing at a red light or not.”*

Also, persons with dementia might view product use as reducing their independence due to feelings of being tracked. Similarly, care partners could feel uneasy when using products due to their tracking nature.

P5, Business, Marketing analyst: *“The persons that wears the product can feel like they are being tracked, and that’s not a good feeling.*

P5, Business, Marketing analyst*: “But also care partners that use the product can feel uneasy because they are tracking persons with dementia.”*

However, professionals pointed out that most care partners feel morally responsible to monitor and that devices offer more ethical forms of monitoring compared to alternative methods such as restricting ambulation.

P6, Business, Company executive: *I see monitoring also positively. There are a lot of people in professional care settings or care partners who feel responsible in providing this monitoring.”*

P6, Business, Company executive: *“If you don’t have such a system, then you have someone telling persons with dementia: “Stop” Stay put! Where are you going again?”*

### Adoption barriers

We identified six recurrent adoption barrier themes and 18 subthemes, displayed in Table 2, Section 2.

(i) ***Awareness limitations.*** A key theme centered on the low awareness of the existence of locating technologies by end-users. This could be attributed in part to poor knowledge transfers between end-users and professional stakeholders. Business professionals indicated product marketing issues leading to low awareness, such as products being released “*way too early*”.

P14, Research, Postdoc (gerontology): *“I can’t use what I don’t know exists. That’s the main problem I learned after conducting 105 interviews [with persons with dementia and care partners].”*

P2, Business, Company executive: *“My personal opinion: Way too early. End-users don’t know that these products exist.”*

Also, the lack of a readily available overview of commercial products, and limited retail access to products leading to complex purchasing processes for end-users were highlighted.

P21, Research, Research associate (gerontechnology): *“General practitioners don’t have an overview of all commercially available products. The same goes for nursing facilities.”*

P4, Business, Software developer: “*If care partners need it [GPS technology], where do they go? Where can you buy it? You won’t find it in a supermarket or media store! You first have to research it and if you’re not from this line of work, it’s hard [to find information].”*

Furthermore, the low technological affinity of most end-users was expressed by research and healthcare professionals.

P18, Research, Project manager (gerontology): *“There are certainly older adults that are good with technologies, which have smartphones. But there are some older adults that have no experience—that are technology skeptic.”*

(ii) ***Technological limitations.*** Technological limitations causing usage-related difficulties also lead to low adoption by not satisfying the expectation that use could help increase quality of life. Research professionals reported on their experience with products that do not provide reliable and accurate location based on poor network communication issues, frequent hiccups, and product maintenance updates.

P22, Research, Research associate (gerontechnology): *“When one enters an underground parking lot or a building, then you can often pretty much forget about location. The product has to be more than 150% reliable. If not, you can forget it!”*

P22, Research, Research associate (gerontechnology): *“It should be low maintenance … You should be able to locate immediately, without having to wait for updates. And if there’s a discrepancy of a few meters and I’m in the pedestrian zone and there are a lot of people around, it could be that I don’t find someone who is two meters away.”*

Furthermore, the limited functionality of available products and poor battery performance were reported as central technological barriers for all groups.

P5, Business, Marketing analyst: *“Geofencing is one aspect. I would program other intelligent functions, such as integrated temperature recognition. There are maybe other things at persons with dementias’ location that could active an alarm. So I would program intelligent systems.”*

P1, Business, Company executive: *“How long does the battery last? Since our latest update, max two days … ”* [P4, Business, Software developer: *“Max? Yeah, that’s a problem.”*].

(iii) ***Product characteristic limitations.*** Regarding the presented locating device, all groups showed high approval for a watch design. However, professionals emphasized that discrepancies between end-users’ needs and available products would discourage adoption.

P6, Business, Company executive: *“I think I’ve realized that we have to think a lot more from the perspective of end-users. This should always be the starting point and then think about hardware and so forth.”*

P2, Business, Company executive: *“Persons with dementia and care partners are not our primary market group.”*

Specifically, they expressed the concept of “less is more”, and the lack of individual configurations that can adapt to changing healthcare needs with advancing disease severity.

P12, Healthcare, Healthcare manager: *“There is a person with dementia who lives in our nursing facility. He doesn’t leave the grounds without his fanny pack. If you could put the product in his fanny pack and it would still work, that would be ideal.”*

P9, Healthcare, Gerontologist: *“I find it good that there are different functions, such as the emergency and two-way communication. But these functions should be individually customizable, looking at actual severity level and other factors.”*

In addition, they stressed that unsatisfactory and stigmatizing aesthetics due to developing products for heterogeneous populations using a one-size-fits-all design approach or due to technological limitations hinder adoption.

P11, Healthcare, Managing director of AD society: *“Most products are not aesthetically pleasing for females.”*

P1, Business, Company executive: *“The products are too big! We would gladly reduce the size if the technology would allow it … The problem is that you need space for a better battery, for power, for … And so that it’s comfortable to wear, particularly if it’s to be worn on the wrist.”*

Moreover, product affordability and insufficient information on additional costs upon purchase were pivotal barriers.

P1, Business, Company executive: *“The biggest barrier is always the price.”*

P10, Healthcare, Managing director of AD society: *“There’s a cost problem at the moment. Can I afford this? Are there any additional costs once I use it? Products are simply too expensive.”*

(iv) ***Capital investment limitations.*** Business professionals were the only group to express that capital investment limitations impacted the successful development and deployment of high-quality products. They criticized the collection of viewpoints on optimal product characteristics without also advocating for higher capital investments to successfully translate viewpoints to product development.

P1, Business, Company executive: *“There’s no one here [in the other groups] that I know was involved in product development, right? There’s a big discrepancy. We could all say how products should be and what could be done. But you have to have the money to do this … you first have to have the money to invest.”*

Moreover, they argued for a better follow-through from research and development phases to product commercialization.

P1, Business, Company executive: *“There are too many products that are not developed to the end.”*

(v) ***Unclear benefits.*** Several unclear benefits on the value of using locating technologies were discussed. These included end-users not recognizing the need to use products that can aid with spatial orientation deficits, utilizing more trusted locating methods such as involving social network members, and the limited number of studies using a user-centered design to better understand end-users’ needs and preferences with unclear information on clinical effectiveness.

P11, Healthcare, Managing director of AD society: *“Persons with dementia do not see that they need it [locating device]. At most, care partners recognize a need.”*

P5, Business, Marketing analyst: *“I [care partners] might pragmatically get more involved with the [local] community.”*

P21, Research, Research associate (gerontechnology): *“End-users should be more involved [in research and development]. They should test products and then we will better understand what needs to be improved.”*

P2, Business, Company executive: *“There are dementia severities, and then it’s always the question: ‘How long can I [care partners] let persons with dementia move about and use the product [without studies with persons with dementia with different AD severities]?”*

Also, previous negative experiences with devices could yield persistent negative perceptions and hinder adoption despite rapidly improving technological innovations.

P1, Business, Company executive: *“The technology is constantly changing. And those [end-users] who did test it three-four years ago … they had bad experiences. And if it doesn’t work on the first attempt: Next! Forget it!”*

(vi) ***Ethical concerns.*** The balance between products being able to both heighten the autonomous mobility and infringe on the personal privacy of persons with dementia via ubiquitous location control by care partners or third-party tracking firms was at the core of the discussion.

P3, Business, Executive associate: *“I think of tracking firms that collect large amounts of data, secretly collecting information on movement profiles … Do we reduce independence or increase security?”*

However, professionals mentioned that care partners’ sense of moral responsibility to provide security for persons with dementia might encourage the adoption of a security-at-all-costs viewpoint, even if information on the collection of movement data by third parties is confusing due to unclear data security and privacy aspects.

P19, Research, Project manager (gerontology): *“As a person with dementia, I have my autonomy, I have my rights. I might not know that I am being located at a particular time. But for care partners, that’s really not a problem because they have a sense of security. There’s a big difference between medical professionals and care partners, where medical professionals say: ‘That’s an infringement on personal freedom’, and care partners say: ‘I don’t care. I have to know where [person with dementia] is!’”*

In addition, research and business professionals added that changing laws pertaining to legal rights on the location of others hinder adoption via slow product development and commercialization.

P6, Business, Company executive: *“We are very involved with this at the moment. How many movement profiles can be programmed and saved, under which conditions, etc.? This is a very difficult situation at the moment for all businesses involved.”*

P2, Business, Company executive: *“Ultimately, it’s a legal problem with too many unknowns. Are we allowed to do this, to do that? This hinders commercialization. First get approval from a court of law. The external framework could be better. This is one of the main reasons why it [GPS technologies] has not spread so quickly.”*

P15, Research, PhD student (gerontology): *“There are a lot of decisions at the moment on what is allowed regarding locating others.”*

### Services and information dissemination strategies

We identified five recurrent themes on salient services and information dissemination strategies and 15 subthemes, displayed in Table 2, Section 2.

(i) ***Digital autonomy support.*** Efforts to support end-users’ digital autonomy upon product purchase was a key theme. Discussed ways to support digital autonomy included offering installation and product training support. Specific examples included providing at-home installments, product education, web-based automated technical support to allow end-users to search for answers to frequently asked questions and customer support telephone numbers, as well as offering case-management support, where a case manager develops and coordinates a comprehensive plan of services based on end-users’ needs.

P17, Research, Project manager (health services administration): *“That you really have an on-location support that also makes house calls to help one get started with the product.”*

P5, Business, Marketing analyst: *“Case-management service support … If I have a person with deficits, with a certain problem severity, then I can also offer other attractive service support features.”*

P6, Business, Company executive: *“ … for example, that telephone numbers are listed on a website, that frequent questions such as ‘How to install the program’, etc. are provided.”*

However, professionals questioned how the suggested services could be cost-effectively financed.

P2, Business, Company executive*: “But these services have to be affordable and there are simply too many older adults that do not have the financial capacity.”*

(ii) ***Emergency support.*** A second type of service that was discussed centered on support in emergency situations. Professionals in all groups agreed that round-the-clock, external emergency call centers should be available to provide real-time assistance should a person with dementia goes missing or if end-users have more pressing questions.

P17, Research, Project manager (health services administration): *“It’s important to have an emergency support call service that answers whatever question you might have.”*

Still, professionals made it clear that providing quality call centers is fraught with challenges. They cautioned that such services are notoriously expensive to manage and that they require a large personnel base.

P6, Business, Company executive*: “When an alarm is set out, because you have personnel changes every 24 hours, you have to have a lot of people that do this [job]. Who does it on the weekends?”*

P22, Research, Research associate (gerontechnology): *“Support that is available 24/7 … but this has to be financed. That’s also really expensive!”*

(iii) ***Information dissemination actors.*** Professionals also discussed the role of several key actors who could help increase product awareness. Taken together, a multi-actor approach including memory clinics, medical supply stores, general practitioners, governments, and healthcare insurance companies was proposed.

P9, Healthcare, Gerontologist: *“You could involve memory clinics.”*

P?, Healthcare: *“It would be really easy to involve medical supply stores.”*

P17, Research, Project manager (health services administration): “*I think that general practitioners should be involved because they are typically the starting point. There’s a trust-based relationship there.”*

P19, Research, Project manager (gerontology): *“There’s a pilot project in [German city], where the government has set up a counselling center also for technology for older adults … They can advise you there … You can go to them, but they can also go to you.”*

P5, Business, Marketing analyst: *“What we need is support from an established healthcare insurance company that creates a ‘service-support platform’.”*

However, healthcare and research professionals cautioned about the lack of regular follow-ups at memory clinics, as well as time limitations of general practitioners and potentially harming patient–doctor trust relationships.

P9, Healthcare, Gerontologist: *“But persons with dementia come here [memory clinic] at max every six months … ”.*

P22, Research, Research associate (gerontechnology): *“If my general practitioner talks to me about such products, I’d feel like they are trying to sell me something. I don’t go to my general practitioner for that.”*

P19, Research, Project manager (gerontology)*: “GPs are saying: ‘What else are we also supposed to do?’ Who pays for this extra work?”*

(iv) ***Product acquisition.*** Furthermore, professionals discussed which product acquisition methods would allow to best reach end-users, increase product familiarity, and facilitate product financing. Main ideas included promoting retail versus online sales, offering trial periods at low or no cost, and exploring the role of government subsidies in product financing.

P19, Research, Project manager (gerontology): *“At the moment, most products can be bought online. So there’s a lack of vendors with whom older adults can talk to. I think personal talks are extremely important.”*

P12, Healthcare, Healthcare manager: *“For me, it would be a requirement that I can test the product first for two to three weeks without having to pay a big amount for this. Maybe a little fee, but not the entire amount.”*

P5, Business, Marketing analyst: *“In nursing care, there are a lot of government care grants … different financial plans, how you can use these various services.”*

(v) ***Product advertising.*** Lastly, the role of promotional activities centering on product advertising was explored. Key recommendations included ensuring that advertising messaging and visuals are non-stigmatizing and that they utilize a end-user focus. For example, this could be achieved by emphasizing the value of using products to help with optimizing the autonomous mobility of persons with dementia rather than focusing on tracking features.

P11, Healthcare, Managing director of AD society (flyer-feedback): *“I prefer the description on this flyer. It’s simple and contains all you need to know … I see security, quality of life, liberty. The visual presentation is good, and the font size is nice and large. This other flyer is not directed toward persons with dementia, but rather only toward care partners.”*

P3, Business, Executive associate: *“We’ve replaced the word tracking with guardian angel.”*

Another suggestion included displaying a seal of quality from respected research institutions on product advertisements to optimize end-users’ trust in products.

P1, Business, Company executive: *“There have to be institutions. That’s why I’m here today … In the end, the Charité or similar is missing. The stamp from ISO does not suffice. When Charité or German Healthcare Ministry is visible, then there’s a completely different quality level that is achieved.”*

Professionals also expected that advertising materials transparently address key concerns that end-users might have centering on data security, product characteristics such as battery life, and service details such as assistance with emergency situations.

P20, Research, PhD (medical sociology and rehabilitation science): *“It could be a marketing problem … for example, that it’s not clear that it can be avoided that everyone sees my data and locate me. If I don’t know that, I don’t buy it.”*

P5, Business, Marketing analyst (flyer feedback): *“What I still don’t know is whether I have to take the watch off every day and charge it.”*

P6, Business, Company executive: *“Let’s say I receive a message at 4am about my mother and this happens three nights in a row. I’ll be woken up and I can’t really help … What happens then?”*

Furthermore, several examples of advertising platforms that were viewed as being able to reach end-users more effectively were mentioned. Identified platforms were television, magazines, and pharmacies.

P14, Research, Postdoc (gerontology): *“There are probably people that don’t check online for this [GPS product], but rather watch TV. So maybe use TV ads to multiply information.”*

P22, Research, Research associate (gerontechnology): *“I saw an ad in [free magazine with large older adult readership] about a high blood pressure product. I thought that was really good. A magazine that a lot of older adults read—not just persons with dementia and care partners. And the magazines are free. You can just take one.”*

P14, Research, Postdoc (gerontology): *“Maybe there should just be ads placed in pharmacy windows.”*

However, business professionals commented on the lack of financial resources to promote products on platforms that might better reach end-users.

P1, Business, Company executive*: “I don’t produce million-dollar TV ads.”*

## Discussion

This study reports on key barriers to the adoption of locating technologies for use in dementia care, as well as on services and information dissemination strategies to increase adoption. Results are relevant for researchers, healthcare and business professionals, including product designers and developers as they highlight that adoption involves more than the technology and products themselves.

Overall, the professionals in our sample held positive views on the use of locating technologies as a way to increase end-users’ quality of life. However, these technologies also raised ethical concerns since they could be seen as restricting the independence of persons with dementia. Therefore, professionals argued for clear and transparent information on how the data and movement profiles of persons with dementia are saved. These findings resonate with previous publications [14, 22, 39]. The mention of these ethical concerns from business professionals is encouraging as others have argued that ATs developers pay too little attention to the needs of end-users or the “human factor” (p. 77) [40]. Also, participants believed that the need for independence of persons with dementia and care partners’ need to locate their loved ones might outweigh data security concerns, a sentiment expressed by end-users themselves [41]. This finding reinforces the importance of creating opportunities for collaborations between business professionals and end-users to ensure that data security and end-user perspectives are integral to product development.

The discussion of adoption barriers revealed that the interplay between barriers is high. For example, low awareness of the existence of locating technologies by end-users could in part be attributed to unsuccessful communication across relevant stakeholders, with limited research on clinical and cost-effectiveness as a driving factor behind this association. In turn, limited research-validated studies on clinical effectiveness discourages healthcare professionals from recommending locating technologies, and hampers larger societal discourses on their value. Regarding product characteristics, the role of technological innovations to maximize individualization and reduce the risk of stigmatization were stressed. Although not explicitly mentioned by professionals, we add that technological innovations that incorporate prominent patterns of AD-related spatial orientation deficits, such as “dimensions of pattern (lapping, random, or pacing), frequency, [and] boundary transgressions” (p. 99) [8] could help ensure that locating technologies better respond to end-users’ needs, desires, and capabilities.

When discussing services, professionals highlighted that supporting the digital autonomy of end-users to help counteract low technological affinity, as well as building trusting relationships with service providers are essential for adoption. Efforts to support digital autonomy to help persons with dementia age-in-place is a timely topic [42], and several examples on ways to optimize digital autonomy were provided. We maintain that services can address end-users’ low technological experience in real-world scenarios by mimicking clinical study environments where products are typically explained and shortly tested before being used for longer periods of time. Furthermore, while discussing information dissemination strategies, professionals provided several recommendations for promotional activities to increase product awareness. Taken together, they indicated that a multi-stakeholder approach is key and advocated the concept of “meeting consumers where they are” by using traditional sources of information dissemination. Also, they mentioned that offering trial periods could help end-users gain experience with a product and enable UX feedback. Indeed, studies show that end-users are more satisfied with the acquisition of ATs when their opinions are factored into device recommendations [43]. Feedback on the presented advertisement flyers indicated that product marketing has a large room for improvement in terms of content and visuals that can be achieved by creating marketing tools in a process of co-creation between persons with dementia, their care partners and business stakeholders. Similar to recent studies [44, 45], professionals’ stressed the importance of placing end-users at the center of marketing activities to reduce stigmatizing keywords and visuals, as well as to ensure that information on functionality and data security are transparently and adequately addressed.

This study has some limitations. First, although asking professionals about their views on the use of locating technologies for dementia care might have resulted in findings reported elsewhere [20, 46], the perspectives of business professionals are largely lacking in the literature. Second, the use of a convenience sampling technique, which was used to ensure that professionals have sufficient knowledge on the use of locating technologies for dementia care, might have resulted in the collection of viewpoints from persons more positively biased toward the use of these technologies. However, other professional stakeholders [14, 20] similarly report high openness toward the use of ATs in dementia care. Still, the possibility of a positive bias cannot be conclusively ruled out, particularly since 22 out of the 70 professionals contacted agreed to participate. However, as previously mentioned, the focus groups generated rich and diverse viewpoints and low recruitment rates using e-mail is typical [47]. Third, the finding that recommendations regarding product pricing were not explicitly discussed in the third section although purchase cost was mentioned as a pivotal barrier for product adoption can be seen as a limitation. This limitation is not unique to our work, and past research with end-users also generally reports a high range of acceptable purchase costs which typically range from 20 to 100 dollars [44]. Still, the fact that product pricing was not explicitly discussed is one example that points to a larger limitation of this study. Upon closer analysis of professionals’ quotes in the second and third sections of the focus group interview, we find that they are largely opinion-based rather than experience-based. Given that we used purposive sampling and that we encouraged professionals to report on their own experiences, this finding suggests that some of the included professional stakeholders may have limited experience on the use of locating technologies for dementia care. Quite revelatory is that at least one business professional explicitly mentioned that persons with dementia and their care partners are not their primary market group. The development and marketing of locating technologies for dementia care that meets end-users’ needs, preferences and values requires a fundamental paradigm shift insofar as this will take time, and will require a end-user focus and a process of co-creation between end-users and professional stakeholders to optimize long-term product adoption. As mentioned by one business professional, dynamic individuals with the financial capital and drive to embark on this quest are needed. Also, a lack of relevant professional expertise, particularly by business professionals, increases the risk of developing and marketing products that end-users perceive as being stigmatizing. Finally, the fact that the discussion took place a few years ago may be seen as a limitation to the present findings. Yet, from what could be found from the scientific literature, no significant changes in the uptake of locating technologies by end-users has occurred since the study was performed, suggesting that adoption barriers have not been adequately addressed.

The strengths of the present study include its qualitative nature to allow for an in-depth exploration of a complex and multifaceted topic [30], as well as its interdisciplinary nature by bringing together stakeholders with different areas of expertise. While others have argued that “multiple stakeholders with differing philosophical viewpoints slow the development, commercialization and deployment of geriatric technologies” (p. 80) [48], our results do not support this view. A key recommendation based on the results of the current study is to provide opportunities for collaborations between end-users and interdisciplinary stakeholders to support the development and commercialization of scientifically-validated, clinically effective locating technologies for dementia care [49]. Also, and to our knowledge, the inclusion of business professionals is new. Business professionals proved to be particularly helpful in understanding business-related topics and hurdles since they provided more examples of service recommendations, were the only group to mention the role of government subsidies in product financing, as well as capital investment limitations impacting the development and deployment of high-quality products. In addition, studies addressing marketing strategies for locating technologies for dementia care are rare [44] even though marketing aspects play a central role in product adoption [26, 27]. Lastly, the focus on locating technologies can be viewed as a strength as viable solutions to increase adoption are still largely limited to extrapolating findings from a broad range of ATs with various applications.

## Conclusions

In conclusion, this paper resonates with past findings on adoption barriers, and identifies services and information dissemination factors that remain to be adequately addressed before the implementation of locating technologies can truly make a difference in dementia care. The need to improve locating solutions and their adoption has been highlighted by the recent creation of international and interdisciplinary consortiums and networks [50, 51]. Moving forward, collaborations between end-users and professional stakeholders that examine what services end-users find appropriate to increase digital autonomy, and what information dissemination strategies to utilize to effectively reach end-users are steps in the right direction.

## Supplementary Information

**Additional file 1.** Product description of GPS watch and smartphone presented to professionals. Table presenting a description of the GPS watch and smartphone presented to professionals during the focus groups, including product name, picture, dimensions, weight, battery, charging, software, and website of product.

**Additional file 2.** COREQ (COnsolidated criteria for REporting Qualitative research) Checklist. Table presenting information of the focus groups following COREQ Checklist reporting style.

## Data Availability

The authors confirm that the data supporting the findings of this article are available within the article.

## Abbreviations

AD: Alzheimer’s disease
ATs: Assistive technologies
GPS: Global Positioning System
UX: User experience
