# Supplementary material for: Overcoming barriers to the adoption of locating technologies in dementia care: a multi-stakeholder focus group study
Source: BMC Geriatr. 2021 Jun 21;21:378. doi: 10.1186/s12877-021-02323-6 (PMC8218472; doi:10.1186/s12877-021-02323-6)
Supplement: Supplementary file 1 — Additional file 1. Product description of GPS watch and smartphone presented to professionals. Table presenting a description of the GPS watch and smartphone presented to professionals during the focus groups, including product name, picture, dimensions, weight, battery, charging, software, and website of product. [file 12877_2021_2323_MOESM1_ESM.pdf]

Additional file 1. Product description of GPS watch and smartphone presented to professionals.

Name	HIMATIC GPS Uhr Alpha [Himatic GPS watch Alpha]	Thl T6C smartphone
Picture	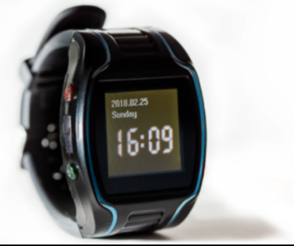	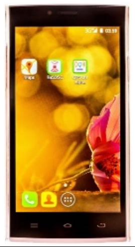
Dimensions	45.5mm X 64.5mm X 17.5mm	71.6mm X 143.9mm X 8.2mm
Weight	70g	160g
Battery	500 mAh, Li-Ion	1900mAh, Li-Ion
Charging	DC 5V USB charger cable	2.0 Micro USB
Software	Native Android App: HIMATIC GPS Uhr Alpha	Android 5.1 Lollipop
Website	https://himaticmobile.de	https://www.thlphone.com/

Abbreviations: GPS, global positioning system; UX, user experience.
NOTE. GPS watch has five buttons and contains a location and telephone function technology. The smartphone application displays the last recognized position of the GPS watch on an online map when prompted. Displayed are two GPS watch apps used in our concurrent user experience study (12), a Google Maps application, a telephone icon, a contacts icon, and a home button.
