# Supplementary material for: Overcoming barriers to the adoption of locating technologies in dementia care: a multi-stakeholder focus group study
Source: BMC Geriatr. 2021 Jun 21;21:378. doi: 10.1186/s12877-021-02323-6 (PMC8218472; doi:10.1186/s12877-021-02323-6)
Supplement: Supplementary file 2 — Additional file 2. COREQ (COnsolidated criteria for REporting Qualitative research) Checklist. Table presenting information of the focus groups following COREQ Checklist reporting style. [file 12877_2021_2323_MOESM2_ESM.pdf]

Additional file 2. COREQ (COnsolidated criteria for REporting Qualitative research) Checklist.

Topic	Description
**Domain 1: Research team and reflexivity**	
Personal characteristics	
Interviewer/facilitator	HM, OP, LW.
Credentials	MSc (Gerontology) / Dr. med. (German medical doctoral degree) / Dr. rer. pol. (German equivalent to PhD in Business Administration).
Occupation	Research associate / Senior physician and academic researcher / Full professor of Information Management and Digital Transformation.
Gender	Female / Male / Male.
Experience and training	Experience in quantitative and qualitative research on assistive technologies, dementia diagnostics, and clinical drug trials in Alzheimer’s disease / Experience in quantitative research, dementia diagnostics, and clinical drug trials in Alzheimer’s disease/ Experience in qualitative research on health-IT and business model innovations.
Relationship with participants	
Relationship established	Conducted previous research with one of the participants in the business group and collaborated with several of the participants in previous Alzheimer’s disease research projects and events / Conducted previous research with one of the participants in the business group and collaborated with several of the participants in previous Alzheimer’s disease research projects and events / No prior knowledge or relationship with any of the participants.
Participant knowledge of the interviewer	Participants received the information that the authors were conducting a research project on assistive technologies in the field of dementia care with a special focus on locating technologies. The communicated goal was to explore the needs of persons with dementia and their caregivers with regards to locating technologies, as well as to explore how these needs could be effectively meet through business model designs.
Interviewer characteristics	Participants received the information that the authors were interested in exploring potential barriers to the adoption of assistive technologies, including locating technologies, in dementia care.
**Domain 2: Study design**	
Theoretical framework	
Methodological orientation and theory	Qualitative description.
Participant selection	
Sampling	Purposive sampling.
Method of approach	Personalized e-mail.
Sample size	22 professionals separated into groups based on their professional field (business, n = 7, healthcare, n = 6, and research, n = 9).
Non-participation	70 professionals were contacted to participate (n = 35, no response, n = 8, unavailable for various reasons, and n = 5, no-show—i.e., agreed to participate, but were not present for the focus group).
Setting	
Setting of data collection	Memory Clinic of the Charité Universitätsmedizin Berlin. Participants were gathered in a conference room to receive a welcome reception and information on organizational details prior to the focus groups, and then separated into one of three rooms for the focus groups.
Presence of non-participants	Silka Dawn Freiesleben (SDF), Valentina Lüdtke (VL), Robert Sonneschein (RS), Gökhan Ozer (GO) and Florian Konwischer (FK) acted as assistants to the researchers.
Description of sample	Yes—see Table 1.
Data collection	
Interview guide	SDF, HM, OP and LW wrote the questions included in the interview guide, and prompts were given during the interviews if needed. No pilot testing.
Repeat interviews	No.
Audio/visual recording	Audio recording performed by SDF, VL; RS, GO and FK, and verbatim transcription performed by SDF, HM, Christina Herrmann (CH).
Field notes	Yes.
Duration	Approximately three hours (including administration of informed consent and filling out of questionnaires).
Data saturation	Assumed to be reached with a sample size of ten to fifteen participants per group based on sample homogeneity.
Transcripts returned	No.
**Domain 3: Analysis and findings**	
Data analysis	
Number of data coders	Three (SDF, HM and CH).
Description of the coding tree	Three main themes and nine subthemes reported in the first section of the focus groups, six main themes and eighteen subthemes reported in the second section of the focus groups, and five main themes and fifteen subthemes reported in the third section of the focus groups—see Table 2.
Derivation of themes	Using content analysis, data-derived themes were identified following an inductive data analysis approach.
Software	MAXQDA.
Participant checking	No.

Reporting	
Quotations presented	Yes, quotations were identified and are presented—see Table 2.
Data and findings consistent	Yes.
Clarity of major themes	Yes—three, six, and five main themes are reported in the first, second, and third sections of the focus groups, respectively.
Clarity of minor themes	Yes—nine, twenty-two, and twenty-three subthemes are reported in the first, second, and third sections of the focus groups, respectively.

NOTE. The information displayed under the header "*Personal characteristics*" regarding credentials, occupation, experience and training, and relationship established reflect the information available at the time of the study.
